# Benefits and risks of antiplatelet medication in hemodynamically stable adult moyamoya disease

**DOI:** 10.1038/s41598-021-99009-1

**Published:** 2021-09-29

**Authors:** Chang Hwan Pang, Won-Sang Cho, Hyun-Seung Kang, Jeong Eun Kim

**Affiliations:** Department of Neurosurgery, Seoul National University Hospital, 101 Daehak-Ro, Jongno-Gu, Seoul, 03080 Republic of Korea

**Keywords:** Neuroscience, Neurology

## Abstract

Revascularization surgery is considered a standard treatment for preventing additional stroke in symptomatic moyamoya disease (MMD). In hemodynamically stable, and asymptomatic or mildly symptomatic patients, however, the treatment strategy is controversial because of the obscure natural course of them. The authors analyzed the benefits and risks of antiplatelet medication in those patients. Medical data were retrospectively reviewed in 439 hemispheres of 243 patients with stable hemodynamic status. Overall, 121 patients (49.8%) with 222 studied hemispheres (50.6%) took antiplatelet medication. Symptomatic cerebral infarction and hemorrhage occurred in 10 (2.3%) and 30 (6.8%) hemispheres, over a mean follow-up of 62.0 ± 43.4 months (range 6–218 months). The use of antiplatelet agents was statistically insignificant in terms of symptomatic infarction, hemorrhage and improvement of ischemic symptoms. In subgroup analyses within the antiplatelet group according to drug potency and duration of medication, a longer duration of antiplatelet medication significantly improved ischemic symptoms (adjusted OR 1.02; 95% CI 1.01–1.03; p = 0.006). Antiplatelet medication failed to prevent symptomatic cerebral infarction or improve ischemic symptoms. However, antiplatelet therapy did not increase the risk of cerebral hemorrhage.

## Introduction

Moyamoya disease (MMD) is an idiopathic progressive steno-occlusive cerebrovascular disease^1^. Hemodynamic insufficiency is a major pathophysiology in MMD, and microembolism is considered one of the causes of ischemic stroke^1–6^. Common clinical manifestations include cerebral ischemia in 50–76% of the patients and hemorrhage in 5–40%^2,7–9^. Revascularization surgery is accepted as an effective treatment to prevent additional stroke in symptomatic patients with ischemic and hemorrhagic MMD^10,11^. In hemodynamically stable adult MMD, optimal management considering perioperative complications and the natural course of the disease is still controversial. For patients with atherosclerotic cerebrovascular diseases^12^, antiplatelet agents have long been used, but there are few studies on the effect or risk of antiplatelet medication in MMD^13–16^. The authors performed a retrospective study to determine whether antiplatelet agents were useful to prevent symptomatic cerebral infarction and to improve ischemic symptoms and whether those agents were safe and caused no cerebral hemorrhage in hemodynamically stable adult MMD.

## Methods

### Patient selection

Medical data of the patients diagnosed with adult MMD between January 2003 and June 2018 were retrospectively reviewed under the approval of the institutional review board and the patient’s informed consent was waived. Inclusion criteria were as follows: (1) age ≥ 20 years; (2) compatibility with the diagnostic guidelines of the MMDs^4,5,7^; (3) an initial clinical follow-up without surgery of at least 6 months or more; (4) initially mild or fixed symptoms related to hemorrhage and ischemia, or asymptomatic presentation; (5) hemodynamically stable status on single-photon-emission computed tomography (SPECT); and (6) medical records pertaining to the evaluation of certain concomitant medical conditions at the first visit and during the follow-up period. All the patients were initially evaluated with SPECT, magnetic resonance imaging and/or digital subtraction angiography. Hemodynamically stable status was defined as a normal or mild decrease in basal perfusion and a decrease in the reserve capacity after acetazolamide challenge at less than 50% of the basal perfusion on basal and acetazolamide-challenged SPECT with 99mTc-hexamethylpropyleneamine oxime^10^. SPECT and/or magnetic resonance perfusion imaging with arterial spin labelling and fluid attenuated inversion recovery are usually followed-up every 2 years during the follow-up, and whenever ischemic symptoms and signs are aggravated.

**Figure 1:**
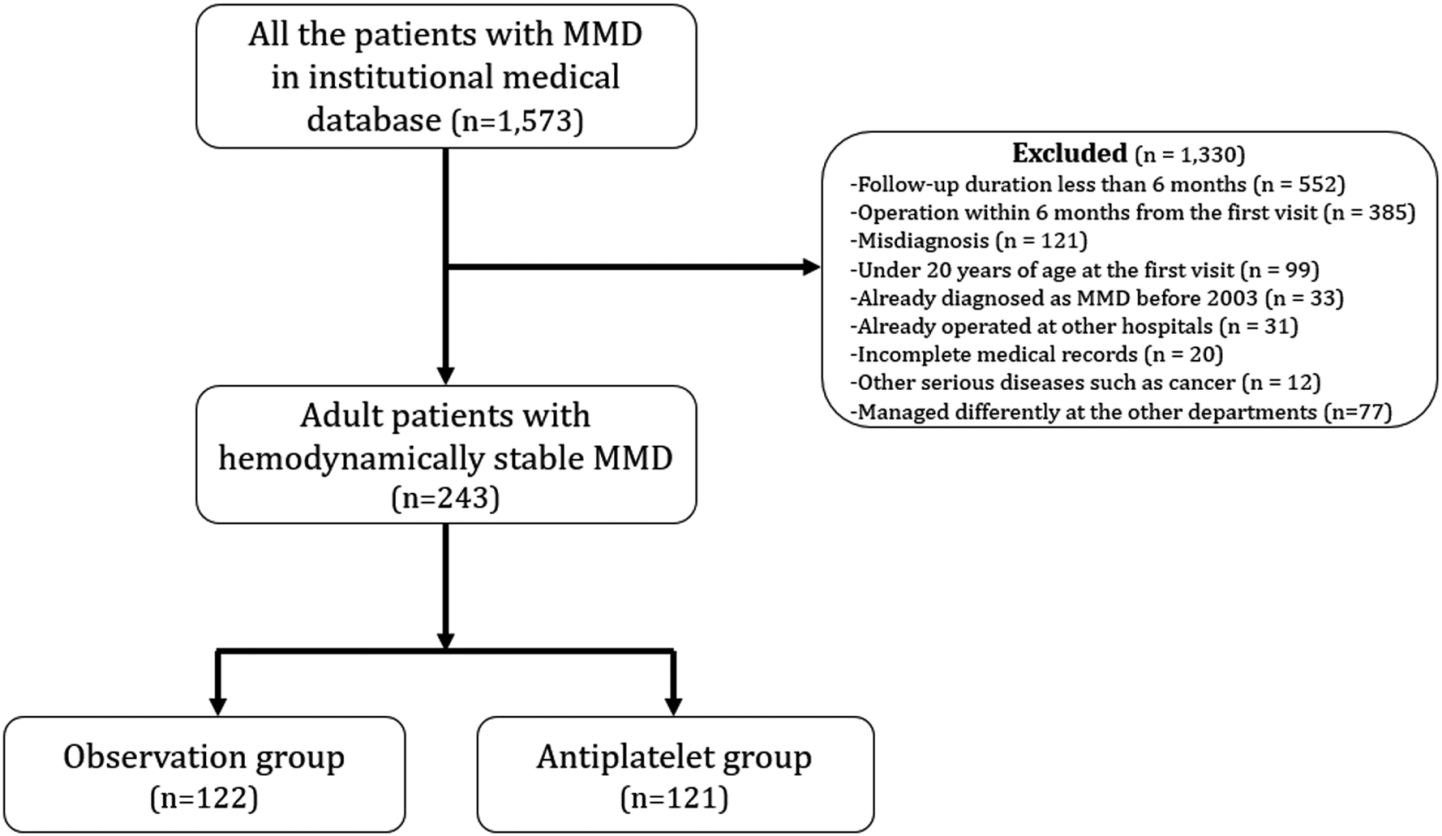
Flow chart of the patient selection. *MMD* moyamoya disease.

A total of 439 hemispheres of 243 patients were finally included (Fig. 1), consisting of 217 hemispheres in 122 patients (observation group) and 222 hemispheres in 121 patients (antiplatelet group). Baseline characteristics are presented in Table 1.

**Table 1:** Baseline characteristics.

Variables	Total	Observation group	Antiplatelet group	p value
Patients/hemispheres, n (%)	243/439	122 (50.2)/217 (49.4)	121 (49.8)/222 (50.6)	
Female, n (%)	182 (74.9)	94 (77.0)	88 (72.7)	0.437
Age, mean ± SD (range), years	43.7 ± 11.4 (20–74)	43.3 ± 10.7 (20–67)	44.2 ± 12.0 (20–74)	0.541
Unilateral MMD, n (%)	47 (19.3)	27 (22.1)	20 (16.5)	0.269
Familial MMD, n (%)	47 (19.3)	25 (20.5)	22 (18.2)	0.649
Current smoking, n (%)	14 (5.8)	3 (2.5)	11 (9.1)	**0.027**
Hypertension, n (%)	85 (35.0)	36 (29.5)	49 (40.5)	0.073
Hyperlipidemia, n (%)	92 (37.9)	32 (26.2)	60 (49.6)	**< 0.001**
**Initial presentation, n**^**a**^** (%)**				
Cerebral ischemia	198 (45.1)	88 (40.6)	110 (49.5)	0.058^†^
Cerebral hemorrhage	46 (10.5)	31 (14.3)	15 (6.8)	**0.010**^†^
Incidental finding	191 (43.5)	98 (45.2)	93 (41.9)	0.432^†^
Other symptoms	5 (1.1)	0	5 (2.3)	0.061^†^
**Perfusion status on SPECT, n**^**a**^** (%)**				
Decrease in basal perfusion	173 (39.4)	79 (36.4)	94 (42.3)	0.203^†^
Decrease in reserve capacity	193 (44.0)	95 (43.8)	98 (44.1)	0.939^†^
**Existence of collaterals, n**^**a**^** (%)**				
Lenticulostriate	125 (28.5)	71 (32.7)	54 (24.3)	0.051^†^
Thalamic	99 (22.6)	53 (24.4)	46 (20.7)	0.353^†^
Choroidal	109 (24.8)	59 (27.2)	50 (22.5)	0.258^†^

p < 0.05 statistically significant (marked in bold).

Unless specified otherwise, values indicate the number or data of patients.

*MMD* moyamoya disease, *SPECT* single photon emission computed tomography.

^†^Logistic regression model using GEE.

^a^Number of hemispheres.

### Antiplatelet medication

Antiplatelet drugs are not routinely prescribed for patients with MMD but are selectively used in this institution for patients presenting with acute cerebral infarction, with repeated ischemic symptoms despite stable hemodynamics^3,5,6^. Some patients with hemorrhagic and asymptomatic MMD to whom antiplatelet agents were prescribed for other medical problems were also included in this study.

Antiplatelet agents were classified into 4 types based on their relative potency. Potency 1 meant weak potency, such as for pentoxifylline, ibudilast and triflusal^17–20^. Potency 2 meant moderate potency, such as for acetyl salicylic acid, cilostazol, dipyridamole and sarpogrelate^19,21,22^. Potency 3 meant strong potency, including for clopidogrel and ticagrelor^23,24^. Finally, Potency 4 meant the strongest drug combinations, in which all the combinations were dual and including at least one drug of Potency 3^25^. For convenience, patients who did not take any antiplatelet agents during the follow-up included in this study were put into the Potency 0 group. Forty-four (13.7%) patients took antiplatelet agents of Potency 1, 171 (53.1%) Potency 2, 68 (21.1%) Potency 3 and 39 (12.1%) Potency 4. Mean duration of antiplatelet drug medication was 72.5 ± 48.4 months (range7–218 months).

### Clinical and radiological evaluation

The initial clinical presentation was divided into ischemic symptoms (transient ischemic attack [TIA] and cerebral infarction), cerebral hemorrhage (intracerebral, intraventricular, subdural and subarachnoid), and incidental findings (no symptoms or nonspecific symptoms such as headache and dizziness). The starting and ending neurological states were evaluated with the modified Rankin scale^26^. Changes in the initial TIA symptoms were checked during the follow-up by evaluating the frequency and intensity of TIA symptoms and were classified into improved, unchanged and aggravated.

For the analysis of risk factors, some medical comorbidities were initially considered, including hypertension, dyslipidemia, diabetes mellitus, hypertension, heart disease, thyroid diseases, hematological diseases, familial history of MMD and current smoking. Among them, only a few factors were selected, and the other factors, due to their low incidence, were excluded to simplify the analysis. Patients who confirmed smoking during any of the follow-up period were defined as current smokers.

Transmedullary collaterals are well-known risk factors for cerebral hemorrhage and are usually composed of lenticulostriate, thalamic and choroidal collaterals^27,28^. When a collateral is dilated or extended beyond the normal territory, that collateral is defined as being present. All of them were evaluated on digital subtraction angiography.

### Statistical analysis

Continuous variables are presented as mean ± SD. Statistical analysis was performed using Student’s *t*-test for continuous variables, while the χ^2^ test or Fisher’s exact test was used for categorical variables. Logistic regression analysis was used to identify the causative variables of symptomatic infarction, symptomatic hemorrhage and improvement of ischemic symptoms. Fine and Gray’s model was used to confirm whether antiplatelet medication affected cerebral infarction or hemorrhage. In addition, a robust sandwich covariance matrix estimate was used considering the correlated data when both hemispheres were evaluated. Since the duration of antiplatelet medication varied from patient to patient, it was considered a time-dependent covariate. To confirm the effects of antiplatelet agents on the improvement of TIA symptoms, multinomial logistic regression analysis was done using generalized estimating equations considering the associations of measurements between the hemispheres of each patient. Data are reported as ORs, HRs, 95% CIs, and two-sided p values. p < 0.050 was considered significant. SAS statistical software (version 9.4; SAS Institute incorporation, Cary, North Carolina, USA) was used.

## Results

### Clinical results

Clinical results are summarized in Table 2. The clinical states in the two groups were similar at the first and last check-up periods. During the follow-up, symptomatic cerebral infarction and hemorrhage occurred in 10 (2.3%) and 30 (6.8%) hemispheres, respectively. Among 198 hemispheres initially presenting with ischemic symptoms, 72.2% (n = 143) improved or remained unchanged during the mean follow-up of 62.0 ± 43.4 months (range 6–218 months). There were no differences between the two groups in terms of clinical status, symptomatic events or changes in ischemic symptoms, except the follow-up duration.

**Table 2:** Clinical results.

Variables	Total	Observation group	Antiplatelet group	p value
**Modified Rankin Scale, mean ± SD (range)**				
Initial	0.6 ± 1.1 (0–5)	0.6 ± 1.2 (0–5)	0.6 ± 1.0 (0–4)	0.879
Last	0.5 ± 1.2 (0–6)	0.4 ± 1.1 (0–5)	0.6 ± 1.3 (0–6)	0.186
**Symptomatic event during follow-up, n**^**a**^** (%)**				
Cerebral infarction	10 (2.3)	5 (2.3)	5 (2.3)	1.000^†^
Cerebral hemorrhage	30 (6.8)	17 (7.8)	13 (5.9)	0.412^†^
**Change of ischemic symptoms, n**^**a**^** (%)**				
Improved	63 (31.8)	29 (33.0)	34 (30.9)	0.099^‡^
Unchanged	80 (40.4)	41 (46.6)	39 (35.5)	
Aggravated	55 (27.8)	18 (20.5)	37 (33.6)	
Follow-up duration, mean ± SD (range), months	62.0 ± 43.4 (6–218)	51.5 ± 35.0 (6–177)	72.5 ± 48.4 (7–218)	**< 0.001**

p < 0.05 statistically significant (marked in bold).

Unless specified otherwise, values indicate the data of patients.

^†^Logistic regression model using GEE.

^‡^Multinomial logistic regression model using GEE.

^a^Number of hemispheres.

### Symptomatic cerebral infarction

A total of 10 symptomatic cerebral infarctions occurred during the follow-up, consisting of 5 events (2.3%) in the observation group and 5 (2.3%) in the antiplatelet group. Univariate analysis showed an insignificant effect of antiplatelet medication (unadjusted HR 1.16; 95% CI 0.30–4.47; p = 0.830). In multivariate analysis, antiplatelet therapy failed to show a preventive effect on cerebral infarction (adjusted HR 1.17; 95% CI 0.27–5.18; p = 0.835; Table 3).

**Table 3:** Logistic regression analysis of symptomatic cerebral infarction associated with antiplatelet medication during the follow-up.

Variables	Adjusted HR	95% CI	p value
Antiplatelet medication	1.20	0.25–5.76	0.835
Female	2.12	0.29–15.68	0.462
Age	0.98	0.91–1.04	0.437
Cerebral ischemia as initial presentation	1.71	0.37–7.82	0.491
Cerebral hemorrhage as initial presentation^a^	NA	NA	NA
Familial MMD	1.74	0.42–7.24	0.449
Current smoking	3.17	0.21–46.36	0.404
Hypertension	2.71	0.57–12.93	0.211
Hyperlipidemia	1.14	0.33–3.88	0.836
Decrease in basal perfusion on SPECT	0.40	0.07–2.35	0.310
Decrease in reserve capacity on SPECT	0.66	0.19–2.38	0.530

p < 0.05 statistically significant.

*NA* not available, *MMD* moyamoya disease, *SPECT* single photon emission computed tomography.

^a^Event number was 0 and not estimated.

### Symptomatic cerebral hemorrhage

A total of 30 symptomatic cerebral hemorrhages occurred during the follow-up, consisting of 17 events (7.8%) in the observation group and 13 (5.9%) in the antiplatelet group. Univariate analysis showed a significant protective effect of antiplatelet medication on symptomatic cerebral hemorrhage (unadjusted HR: 0.44; 95% CI 0.20–0.99; p = 0.046). In multivariate analysis, however, antiplatelet therapy failed to show a preventive effect on cerebral hemorrhage (adjusted HR 1.19; 95% CI 0.24–1.34; p = 0.193; Table 4). Interestingly, the existence of choroidal collaterals significantly increased the risk of cerebral hemorrhage (adjusted HR 2.84; 95% CI 1.31–6.18; p = 0.008).

**Table 4:** Logistic regression analysis of symptomatic cerebral hemorrhage associated with antiplatelet medication during the follow-up.

Variables	HR	95% CI	p value
Antiplatelet medication	0.56	0.24–1.34	0.193
Female	1.57	0.35–7.09	0.556
Age	1.01	0.98–1.04	0.654
Cerebral ischemia as initial presentation	0.57	0.20–1.62	0.292
Cerebral hemorrhage as initial presentation	0.86	0.27–2.72	0.795
Familial MMD	1.49	0.67–3.31	0.332
Current smoking	4.05	0.54–30.60	0.176
Hypertension	0.53	0.22–1.24	0.141
Hyperlipidemia	1.07	0.50–2.29	0.857
Decrease in basal perfusion on SPECT	1.22	0.58–2.60	0.600
Decrease in reserve capacity on SPECT	0.99	0.43–2.28	0.981
Lenticulostriate collaterals	1.42	0.57–3.54	0.449
Thalamic collaterals	1.52	0.63–3.65	0.354
Choroidal collaterals	2.84	1.31–6.18	**0.008**

p < 0.05 statistically significant (marked in bold).

*MMD* moyamoya disease, *SPECT* single photon emission computed tomography.

### Improvement of ischemic symptoms

In a total of 198 hemispheres of 116 patients who initially presented with ischemic symptoms, 63 hemispheres (31.8%) showed an improvement in ischemic symptoms. Antiplatelet medication failed to show a positive effect on the improvement of ischemic symptoms in the univariate (unadjusted OR 0.95; 95% CI 0.50–1.81; p = 0.876) or multivariate analysis (adjusted OR 0.80; 95% CI 0.40–1.62; p = 0.541) (Table 5).

**Table 5:** Logistic regression analysis of improvement of ischemic symptoms associated with antiplatelet medication during the follow-up.

Variables	Adjusted HR	95% CI	p value
Antiplatelet medication	0.80	0.40–1.62	0.541
Female	1.16	0.52–2.60	0.711
Age	1.01	0.97–1.04	0.775
Familial MMD	0.50	0.19–1.29	0.150
Current smoking	3.98	0.71–22.40	0.117
Hypertension	0.64	0.29–1.41	0.270
Hyperlipidemia	1.91	0.91–4.02	0.089
Decrease in basal perfusion on SPECT	1.72	0.89–3.33	0.108
Decrease in reserve capacity on SPECT	0.63	0.34–1.17	0.144

p < 0.05 statistically significant.

*MMD* moyamoya disease, *SPECT* single photon emission computed tomography.

### Subgroup analysis within the antiplatelet group

In terms of symptomatic cerebral infarction, neither drug potency nor duration of medication had a preventive effect (Supplementary Tables 1 and 2). In terms of symptomatic cerebral hemorrhage, the potency of antiplatelet agents was insignificant (Supplementary Table 3), and the duration of medication was of borderline significance (adjusted HR 0.99; 95% CI 0.97–0.100; p = 0.085). In the multivariate analysis of the duration of medication, however, cerebral ischemia at the initial presentation was a significant preventive factor against cerebral hemorrhage (adjusted HR 0.07; 95% CI 0.01–0.77; p = 0.030; Supplementary Table 4). Finally, in terms of the improvement of ischemic symptoms, a longer duration of antiplatelet medication (adjusted OR 1.02; 95% CI 1.01–1.03; p = 0.006) and current smoking (adjusted OR 19.05; 95% CI 1.27–286.87; p = 0.033) showed positive effects (Supplementary Table 5).

## Discussion

Surgical revascularization is well known to be effective in preventing recurrent stroke in adult MMD^4,10,11^. In patients with hemodynamically stable but clinically symptomatic MMD, however, effective medical management has long been debated, weighing unignorable natural course against surgical burdens^5,8,10,11,13–16,29,30^. Disappointingly, in this study, the use of antiplatelet agents had no significant effect in the prevention of cerebral infarction, improvement of initial ischemic symptoms or occurrence of cerebral hemorrhage. The existence of choroidal collaterals was a significant risk factor for cerebral hemorrhage, which has recently become well known^27,28^. In the subgroup analysis within the antiplatelet group, duration of antiplatelet medication and current smoking were significant factors improving the initial ischemic symptoms. The positive result of current smoking should be cautiously interpreted, considering the wide 95% CI.

The natural history of MMD varies slightly according to the ethnicity, institution and status of the patient group. Patients with some characteristics, such as Asian ethnicity, symptomatic presentation and a low perfusion status, are reported to show a higher risk of stroke^8,29,31–33^, and these are usually considered surgical indications for the prevention of recurrent bleeding and infarction^11,29^. Asymptomatic or hemodynamically stable symptomatic patients seem to have a benign clinical course; however, they are not in a stable status, given their annual stroke risk of 3.2–4.5%^8,33^. Meanwhile, perioperative complications occur often (cerebral hemorrhage in 3.1%; infarction in 4.0%), so that at least 2 or 3 years are needed to overcome the natural course of disease in conservatively managed patients^10,29,30^. Therefore, physicians always feel a strong urge to find effective medical management for hemodynamically stable patients with asymptomatic or mildly symptomatic presentations.

Antiplatelet agents have long been used for primary and secondary prevention of stroke and are backed by concrete evidences^12^. They are frequently prescribed for MMD patients presenting with ischemic symptoms in the real world^34,35^, and antiplatelet medication is recommended in the recent guidelines for MMD, although the level of evidence is not high^5^. The major pathomechanism of MMD is hemodynamic insufficiency caused by progressive steno-occlusion of the intracranial arteries^1,2,4,5^. However, as intraluminal microthromboembolism was recently shown to be one of the ischemic symptoms in patients with MMD^3,6^, the use of antiplatelet agents is increasing and is expected to inhibit platelet aggregation and thrombus formation. Moreover, cilostazol is thought to improve hemodynamic insufficiency through a vasodilatory effect as one of its pleiotropic effects^15^. Antiplatelet medication is selectively used in this institution for patients presenting with repeated ischemic symptoms and signs despite stable hemodynamics, a situation probably caused by microembolism. However, there still exists a major risk of antiplatelet agents to look into, intracranial hemorrhage^21^, though there is no definite evidence on this connection.

A few studies about the effects of antiplatelet medication have been recently reported in patients with MMD^13–16^. Their preventive effect on ischemic stroke is not consistent, though antiplatelet agents do not seem to increase the risk of cerebral hemorrhage. However, those studies have some limitations, such as not mentioning the kinds of drugs used or the indications of drug administration and the inhomogeneous basal statuses of the patients. A prospective observational study reported that antiplatelet agents did not reduce recurrent cerebral infarction, whereas the rate of cerebral hemorrhage was significantly lower in the antiplatelet group^13^. However, the results were derived from the univariate analysis within the ischemic subgroup alone. A retrospective propensity score-matched study demonstrated that patients who received prehospital antiplatelet agents had a significantly better initial status and outcome^14^. In a propensity score-matched study of national health data^15^, any use of antiplatelet agents reduced the mortality rate, and cilostazol showed the best effect. A recent propensity score-matched study in adult ischemic MMD showed that the risk of recurrent ischemic attack was lowest in the surgery group and that the antiplatelet group also had lower recurrence rates than the observation group, with no differences in bleeding events or functional outcomes^16^. However, treatment modality was determined only based on the patients’ clinical states and their choice.

In this study, the use of antiplatelet agents did not influence the occurrence of cerebral infarction, hemorrhage and the improvement of initial ischemic symptoms. Patients visiting this medical team are evaluated according to the predetermined work-up protocol, and the treatment strategy is determined based on the test findings and indications this team has established^4,8,10,36,37^. Therefore, it is thought that the patients included in this study would be more homogeneous than those in previous reports. This study has some limitations, such as its retrospective nature, lack of randomization and use of a few kinds of antiplatelet agents^13–16^. Interestingly, in a search of the institutional database, the authors found not a few adult MMD patients who were managed by the other teams of the same institution. Analyzing of a total of 439 hemispheres in 243 patients in the same period, antiplatelet medication was insignificant in terms of cerebral infarction, hemorrhage and improvement of ischemic symptoms. In the subgroup analysis within the antiplatelet group of 110 hemispheres in 66 patients, however, a longer duration of antiplatelet medication significantly reduced symptomatic cerebral infarction and hemorrhage. Such different results are thought to originate from the inhomogeneous basal characteristics of the patients included in the additional analysis because the management strategies are very different among the medical teams. Thus, a study design with a clear definition of the patient group is very important for obtaining valuable results, and this should be considered when designing prospective studies in the near future. As revascularization surgery for hemodynamically unstable MMD is definitely effective in the prevention of recurrent events^10,13^, surgery is judged to be more beneficial than antiplatelet medication in unstable patients despite the benefits of antiplatelet medication and the perioperative complications. In hemodynamically stable, and asymptomatic or mildly symptomatic patients, antiplatelet medication could be tried, considering the natural course of those patients and their conditions^8,13^, because the risk of antiplatelet agents seems insignificant and drugs are effective in certain patients. However, choroidal collaterals are a recent well-known risk factor of rebleeding and de novo bleeding in MMD^27,28^, which was also proved in this study. Although antiplatelet medication was insignificant in bleeding event in this study, it is necessary to be more careful in prescribing antiplatelet agents in patients with choroidal collaterals.

In conclusion, antiplatelet medication in adult patients with hemodynamically stable MMD did not show any benefits or risks in the occurrence of symptomatic stroke or improvement of initial ischemic symptoms, despite other significant factors related to cerebral hemorrhage or the improvement of ischemic symptoms in the subgroup analysis. Well-designed prospective studies with some drugs widely used and recently known to be effective, such as aspirin, clopidogrel and cilostazol^15,38^, are expected to overcome the limitations of previous studies, including this, and to suggest more concrete answers.

## Supplementary Information

Supplementary Information.

## Data Availability

The data are available from the corresponding author on reasonable request approved the institutional review boards of Seoul National University Hospital.
