# Supplementary material for: Benefits and risks of antiplatelet medication in hemodynamically stable adult moyamoya disease
Source: Sci Rep. 2021 Sep 29;11:19367. doi: 10.1038/s41598-021-99009-1 (PMC8481560; doi:10.1038/s41598-021-99009-1)
Supplement: Supplementary file 1 — Supplementary Information. [file 41598_2021_99009_MOESM1_ESM.docx]

**Supplementary table 1.** Logistic regression analysis of symptomatic cerebral infarction associated with the potency of antiplatelet agents during the follow-up

| **Univariate analysis** | | | |
| --- | --- | --- | --- |
| **Variables** | **Unadjusted HR** | **95% CI** | **P value** |
| Potency |  |  |  |
| 0 vs 4^*^ | NA | NA | NA |
| 1 vs 4^*^ | NA | NA | NA |
| 2 vs 4 | 0.44 | 0.05 to 4.36 | 0.485 |
| 3 vs 4 | 0.73 | 0.07 to 7.63 | 0.792 |
| **Multivariate analysis** | | | |
| **Variables** | **Adjusted HR** | **95% CI** | **P value** |
| Potency |  |  |  |
| 0 vs 4^*^ | NA | NA | NA |
| 1 vs 4^*^ | NA | NA | NA |
| 2 vs 4 | 0.40 | 0.05 to 3.63 | 0.418 |
| 3 vs 4 | 0.76 | 0.06 to 9.96 | 0.837 |
| Female | 1.25 | 0.12 to 12.97 | 0.853 |
| Age | 0.96 | 0.82 to 1.11 | 0.542 |
| Cerebral ischemia as initial presentation | 0.29 | 0.05 to 1.75 | 0.177 |
| Cerebral hemorrhage as initial presentation^*^ | NA | NA | NA |
| Familial MMD | 3.78 | 0.25 to 57.31 | 0.338 |
| Current smoking^*^ | NA | NA | NA |
| Hypertension | 0.37 | 0.04 to 3.57 | 0.387 |
| Hyperlipidemia | 4.13 | 0.49 to 35.05 | 0.194 |
| Decrease in basal perfusion on SPECT | 1.80 | 0.15 to 21.01 | 0.640 |
| Decrease in reserve capacity on SPECT | 0.84 | 0.15 to 4.68 | 0.843 |

p<0.05 statistically significant

^*^Event number was 0 and not estimated.

NA, not available; MMD, moyamoya disease; SPECT, single photon emission computed tomography.

**Supplementary table 2.** Logistic regression analysis of symptomatic cerebral infarction associated with the duration of antiplatelet medication during the follow-up

| **Univariate analysis** | | | |
| --- | --- | --- | --- |
| **Variables** | **Unadjusted HR** | **95% CI** | **P value** |
| Duration of antiplatelet medication | 0.99 | 0.99 to 1.00 | 0.088 |
| **Multivariate analysis** | | | |
| **Variables** | **Adjusted HR** | **95% CI** | **P value** |
| Duration of antiplatelet medication | 1.00 | 0.99 to 1.00 | 0.278 |
| Female | 1.60 | 0.13 to 19.20 | 0.712 |
| Age | 0.96 | 0.87 to 1.06 | 0.444 |
| Cerebral ischemia as initial presentation | 0.48 | 0.12 to 1.86 | 0.288 |
| Cerebral hemorrhage as initial presentation^*^ | NA | NA | NA |
| Familial MMD | 2.14 | 0.22 to 20.63 | 0.512 |
| Current smoking^*^ | NA | NA | NA |
| Hypertension | 0.59 | 0.13 to 2.81 | 0.510 |
| Hyperlipidemia | 7.06 | 0.97 to 51.64 | 0.054 |
| Decrease in basal perfusion on SPECT | 1.56 | 0.13 to 18.79 | 0.724 |
| Decrease in reserve capacity on SPECT | 0.90 | 0.16 to 5.01 | 0.902 |

p<0.05 statistically significant

^*^Event number was 0 and not estimated.

NA, not available; MMD, moyamoya disease; SPECT, single photon emission computed tomography.

**Supplementary table 3.** Logistic regression analysis of symptomatic cerebral hemorrhage associated with the potency of antiplatelet agents during the follow-up

| **Univariate analysis** | | | |
| --- | --- | --- | --- |
| **Variables** | **Unadjusted HR** | **95% CI** | **P value** |
| Potency |  |  |  |
| 0 vs 4 | 1.66 | 0.24 to 11.64 | 0.611 |
| 1 vs 4 | 3.47 | 0.39 to 30.87 | 0.264 |
| 2 vs 4 | 0.79 | 0.10 to 6.13 | 0.824 |
| 3 vs 4^*^ | NA | NA | NA |
| **Multivariate analysis** | | | |
| **Variables** | **Adjusted HR** | **95% CI** | **P value** |
| Potency |  |  |  |
| 0 vs 4 | 0.53 | 0.03 to 8.98 | 0.661 |
| 1 vs 4 | 1.57 | 0.05 to 47.96 | 0.798 |
| 2 vs 4 | 0.33 | 0.02 to 6.39 | 0.459 |
| 3 vs 4^*^ | NA | NA | NA |
| Female | 0.97 | 0.07 to 13.04 | 0.984 |
| Age | 1.04 | 0.98 to 1.09 | 0.208 |
| Cerebral ischemia as initial presentation | 0.08 | 0.00 to 1.65 | 0.101 |
| Cerebral hemorrhage as initial presentation | 0.67 | 0.12 to 3.62 | 0.643 |
| Familial MMD | 3.42 | 0.57 to 20.66 | 0.180 |
| Current smoking | 1.88 | 0.10 to 35.90 | 0.676 |
| Hypertension | 0.48 | 0.07 to 3.10 | 0.439 |
| Hyperlipidemia | 1.41 | 0.31 to 6.41 | 0.661 |
| Decrease in basal perfusion on SPECT | 1.39 | 0.33 to 5.87 | 0.653 |
| Decrease in reserve capacity on SPECT | 5.45 | 0.65 to 45.47 | 0.117 |
| Lenticulostriate collaterals | 3.88 | 0.93 to 16.22 | 0.063 |
| Thalamic collaterals | 2.69 | 0.65 to 11.24 | 0.174 |
| Choroidal collaterals | 2.04 | 0.53 to 7.88 | 0.302 |

p<0.05 statistically significant

^*^Event number was 0 and not estimated.

NA, not available; MMD, moyamoya disease; SPECT, single photon emission computed tomography.

**Supplementary table 4.** Logistic regression analysis of symptomatic cerebral hemorrhage associated with the duration of antiplatelet medication during the follow-up

| **Univariate analysis** | | | |
| --- | --- | --- | --- |
| **Variables** | **Unadjusted HR** | **95% CI** | **P value** |
| Duration of antiplatelet medication | 0.99 | 0.98 to 1.00 | **0.012** |
| **Multivariate analysis** | | | |
| **Variables** | **Adjusted HR** | **95% CI** | **P value** |
| Duration of antiplatelet medication | 0.99 | 0.97 to 1.00 | 0.085 |
| Female | 1.18 | 0.10 to 14.54 | 0.898 |
| Age | 1.04 | 0.98 to 1.09 | 0.200 |
| Cerebral ischemia as initial presentation | 0.07 | 0.01 to 0.77 | **0.030** |
| Cerebral hemorrhage as initial presentation | 0.92 | 0.18 to 4.81 | 0.925 |
| Familial MMD | 2.36 | 0.44 to 12.74 | 0.319 |
| Current smoking | 1.79 | 0.15 to 21.86 | 0.649 |
| Hypertension | 0.49 | 0.10 to 2.49 | 0.390 |
| Hyperlipidemia | 1.66 | 0.58 to 4.78 | 0.347 |
| Decrease in basal perfusion on SPECT | 1.81 | 0.44 to 7.57 | 0.414 |
| Decrease in reserve capacity on SPECT | 5.12 | 0.60 to 43.58 | 0.135 |
| Lenticulostriate collaterals | 3.37 | 0.64 to 17.93 | 0.154 |
| Thalamic collaterals | 2.13 | 0.45 to 10.07 | 0.340 |
| Choroidal collaterals | 1.29 | 0.30 to 5.51 | 0.731 |

p<0.05 statistically significant (marked in bold)

MMD, moyamoya disease; SPECT, single photon emission computed tomography.

**Supplementary table 5.** Logistic regression analysis of improvement of ischemic symptoms associated with the duration of antiplatelet medication during the follow-up

| **Univariate analysis** | | | |
| --- | --- | --- | --- |
| **Variables** | **Unadjusted OR** | **95% CI** | **P value** |
| Duration of antiplatelet medication | 1.01 | 1.01 to 1.02 | **0.003** |
| **Multivariate analysis** | | | |
| **Variables** | **Adjusted OR** | **95% CI** | **P value** |
| Duration of antiplatelet medication | 1.02 | 1.01 to 1.03 | **0.006** |
| Female | 1.08 | 0.31 to 3.76 | 0.899 |
| Age | 1.02 | 0.97 to 1.08 | 0.465 |
| Familial MMD | 0.40 | 0.08 to 2.05 | 0.274 |
| Current smoking | 19.05 | 1.27 to 286.87 | **0.033** |
| Hypertension | 0.41 | 0.13 to 1.29 | 0.127 |
| Hyperlipidemia | 1.69 | 0.56 to 5.10 | 0.353 |
| Decrease in basal perfusion on SPECT | 0.71 | 0.08 to 6.19 | 0.759 |
| Decrease in reserve capacity on SPECT | 0.82 | 0.27 to 2.51 | 0.728 |

p<0.05 statistically significant (marked in bold)

MMD, moyamoya disease; SPECT, single photon emission computed tomography.
